# 6-[(4-Hy­droxy­phen­yl)diazenyl]-1,10-phenanthrolin-1-ium chloride monohydrate

**DOI:** 10.1107/S1600536811044941

**Published:** 2011-11-02

**Authors:** Akram Hazeen, Yan Zhang, Minchong Mao, Kraig A. Wheeler, Mark E. McGuire

**Affiliations:** a Department of Chemistry, 600 Lincoln Ave., Charleston, IL 61920, USA

## Abstract

In the cation of the title mol­ecular salt, C_18_H_13_N_4_O^+^·Cl^−^·H_2_O, the dihedral angle between the mean planes of the 1,10-phenanthroline system and the phenol ring is 14.40 (19)°. The crystal packing is stabilized by O—H⋯O hydrogen bonds, weak N—H⋯Cl and O—H⋯Cl inter­molecular inter­actions and π—π stacking inter­actions [centroid–centroid distance = 3.6944 (13) and 3.9702 (12) Å]

## Related literature

For Ru(II)–polypyridyl complexes as solar energy conversion catalysts, see: Vos & Kelly (2006 ▶). For strongly absorbing Ru(II) complexes containing azo-dye ligands, see: McGuire *et al.* (1998 ▶); Malinowski & McGuire (2003 ▶); For the pK_a_ of the phenol portion of these complexes, see: Zhang (1999 ▶). For the synthesis and characterization of 1,10-phenanthroline­azo­sulfonamide derivatives and their ternary Ni(II) complexes, see: Aly *et al.* (2006 ▶). For the synthesis of 5-nitro-1,10-phenanthroline, see: Amouyal *et al.* (1990 ▶) and of 5-amino-1,10-phenanthroline, see: Nasielski-Hinkens *et al.* (1981 ▶). For the crystal structure of 4-[(*E*)-1-naphthyl­diazen­yl]phenol, see: Aslanov *et al.* (2009 ▶) and of 2-pyridyl-diazo-1,3 phenol, see: Xu *et al.* (1982 ▶).
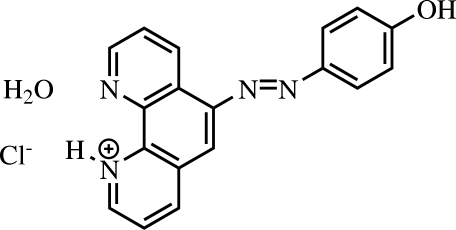


## Experimental

### 

#### Crystal data

C_18_H_13_N_4_O^+^·Cl^−^·H_2_O
*M* *_r_* = 354.79
Triclinic, 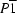

*a* = 7.6732 (4) Å
*b* = 7.7894 (4) Å
*c* = 14.1225 (7) Å
α = 78.535 (3)°
β = 80.379 (3)°
γ = 78.212 (3)°
*V* = 802.73 (7) Å^3^
*Z* = 2
Cu *K*α radiation
μ = 2.28 mm^−1^
*T* = 100 K
0.41 × 0.26 × 0.04 mm

#### Data collection

Bruker APEXII CCD diffractometer
Absorption correction: multi-scan (*SADABS*; Bruker, 2008 ▶) *T*_min_ = 0.454, *T*_max_ = 0.914
15401 measured reflections
2806 independent reflections
2407 reflections with *I* > 2σ(*I*)
*R*_int_ = 0.051

#### Refinement

*R*[*F* ^2^ > 2σ(*F* ^2^)] = 0.043
*wR*(*F* ^2^) = 0.118
*S* = 1.03
2806 reflections
242 parameters
H atoms treated by a mixture of independent and constrained refinement
Δρ_max_ = 0.40 e Å^−3^
Δρ_min_ = −0.21 e Å^−3^

### 

Data collection: *APEX2* (Bruker, 2008 ▶); cell refinement: *SAINT* (Bruker, 2008 ▶); data reduction: *SAINT*; program(s) used to solve structure: *SHELXS97* (Sheldrick, 2008 ▶); program(s) used to refine structure: *SHELXL97* (Sheldrick, 2008 ▶); molecular graphics: *X-SEED* (Barbour, 2001 ▶); software used to prepare material for publication: *SHELXL97*.

## Supplementary Material

Crystal structure: contains datablock(s) I, global. DOI: 10.1107/S1600536811044941/jj2106sup1.cif

Structure factors: contains datablock(s) I. DOI: 10.1107/S1600536811044941/jj2106Isup2.hkl

Supplementary material file. DOI: 10.1107/S1600536811044941/jj2106Isup3.mol

Additional supplementary materials:  crystallographic information; 3D view; checkCIF report

## Figures and Tables

**Table 1:** Hydrogen-bond geometry (Å, °)

*D*—H⋯*A*	*D*—H	H⋯*A*	*D*⋯*A*	*D*—H⋯*A*
O1—H4⋯O2^i^	0.95 (3)	1.65 (3)	2.586 (2)	166 (3)
N2—H19⋯Cl1	0.87 (3)	2.35 (3)	3.1077 (19)	145 (2)
O2—H20⋯Cl1	0.84 (3)	2.25 (3)	3.0959 (16)	180 (3)
O2—H21⋯Cl1^ii^	0.85 (3)	2.30 (3)	3.1467 (17)	170 (2)

Symmetry codes: (i) 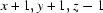; (ii) 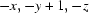.

**Fig. 1.**
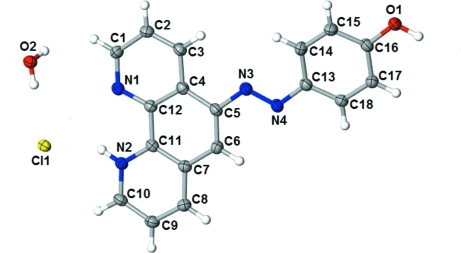
: Perspective view of the title compound, with the atom numbering; displacement ellipsoids are at the 50% probability level.

**Fig. 2.**
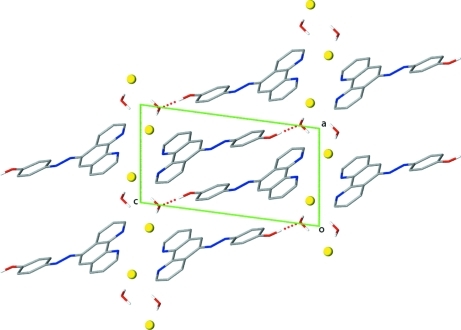
: Packing diagram for the title compound viewed along the ac plane. Dashed lines indicate O1—H4···O2 hydrogen bonds.

## supplementary crystallographic information

### Comment

Ru(II)-polypyridyl complexes have a long history as solar energy conversion catalysts (Vos & Kelly, 2006). The molecular prototype Ru^II^(bpy)_3_^2+^ (bpy = 2,2'-bipyridine) compound and many of its analogs show extremely strong absorption in the 440–500 nm range. This absorption arises from a metal-to- ligand charge transfer (^1^MLCT) transition which relaxes to and populates a charge transfer excited state from which photon emission (typically around 600 nm) or electron transfer can occur. In our prior work in this area, we have been trying to increase the molar absorptivity of these complexes by producing polypyridyl ligands that strongly absorb visible light between 400 and 600 nm and thus act as "antennae" in Ru(II) and related metal complexes (McGuire *et al.*, 1998). Specifically, we have synthesized and characterized a 1,10-phenanthroline-based azo dye ligand (4-[1,10]-phenanthrolinium-1-ium-5-yl-phenol chloride hydrate) consisting of 1,10-phenanthroline bonded at the 5-position to the *para* position on phenol through a diazo linkage. This ligand shows absorption in the 390–544 nm range depending on the solvent and the presence or absence of added acid or base (Malinowski & McGuire, 2003). The pK_a_ of the phenol portion has been measured at 7.6 in water (Zhang, 1999). The following related crystal structures have been reported: 4-[(*E*)-1-naphthyldiazenyl]phenol (Aslanov *et al.*, 2009); 2-pyridyl-diazo-1,3 phenol (Xu *et al.*, 1982).

In the title salt, (I), the ligand crystallized as the monohydrochloride monohydrate (Fig. 1).  Crystal packing is stabilized by O1—H4···O2 hydrogen bonds, weak N2—H19···Cl1, O2—H20···Cl1, O2—H21···Cl1, intermolecular interactions (Table 1), N^+^ protonated cation (1,10-phenanthroline ring)— Cl^-^ anion interactions (Fig. 2) and π—π stacking interactions [centroid-centroid distance = 3.6944 (13)Å (Cg3—Cg4) and 3.9702 (12)Å (Cg1—Cg4); Cg1 = N1/C1—C4/C12, Cg3 = C4/—C7/ C11/C12, Cg4 = C13—C18].

### Experimental

5-Nitro-1,10-phenanthroline (Amouyal *et al.*, 1990) was recrystallized from 95% ethanol and then converted to 5-amino-1,10-phenanthroline (5-NH_2_ phen) (Nasielski-Hinkens *et al.*, 1981). The 5-NH_2_-phen was diazotized by dissolving 0.1962 g (1.006 mmol) in 6 *M* HCl (4 ml). The resulting red solution was immersed in an ice bath and stirred for 2 min. NaNO_2_ (0.0713 g, 1.03 mmol) was dissolved in water (2 ml) and immersed in an ice bath. The NaNO_2_ solution was then added to the 5-NH_2_phen solution and stirred for 3 min. Phenol (0.0950 g, 1.01 mmol) was dissolved in 10 ml of a 10%(*w*/*w*) aqueous solution of NaOH and the solution was stirred for 2 min in an ice bath. This solution was then added to the solution of diazotized phenanthroline. A dark red-orange precipitate formed immediately and the mixture (pH > 10) was left to stir in an ice-bath for 4 h. The pH was adjusted to 6 with 2*M* HCl. The mixture was stirred at room temperature for 30–45 min, and the solid was collected by vacuum filtration and washed with cold water. Yield of dried crude product: 0.2018 g (66.82% based on 5-NH_2_phen). Purification was performed on a 20 *x* 1-cm column of 80–200 mesh alumina (Fisher) that had been slurry- packed using 50:50 CH_2_Cl_2_:*ab* EtOH. A 50.1-mg sample of crude product was dissolved in 25:25:50 *ab*EtOH:MeOH:CH_2_Cl_2_ and filtered on a fine frit. The filtrate was loaded on a column and eluted with 25:25:50 *ab* EtOH:MeOH:CH_2_Cl_2_ resulting in two bands: yellow-orange and pink. The pink band was eluted by MeOH followed by the yellow-orange band. Evaporation of the MeOH and vacuum drying resulted in 32 mg of purified product. Crystals were grown by dissolving a small amount of solid in 1 ml of THF along with one drop of conc. HCl. This mixture was filtered using a Pasteur pipette and glass wool. Crystallization occurred after one week by slow evaporation at room temperature.

### Refinement

H atoms attached to N and O atoms were found in a difference Fourier map and refined independently using isotropic atomic displacement parameters. All of the H atoms bonded to aromatic C atoms were placed in geometrically calculated positions (C—H = 0.95 Å) and were included in the refinement in a riding model approximation, with *U*_iso_(H) = 1.2U_eq_(C).

### Crystal data

**Table:**

C_18_H_13_N_4_O^+^·Cl^−^·H_2_O	*Z* = 2
*M**_r_* = 354.79	*F*(000) = 368
Triclinic, *P*1	*D*_x_ = 1.468 Mg m^−^^3^
Hall symbol: -P 1	Cu *K*α radiation, λ = 1.54178 Å
*a* = 7.6732 (4) Å	Cell parameters from 4384 reflections
*b* = 7.7894 (4) Å	θ = 3.2–66.7°
*c* = 14.1225 (7) Å	µ = 2.28 mm^−^^1^
α = 78.535 (3)°	*T* = 100 K
β = 80.379 (3)°	Transparent plate, orange
γ = 78.212 (3)°	0.41 × 0.26 × 0.04 mm
*V* = 802.73 (7) Å^3^	

### Data collection

**Table:**

Bruker APEXII CCD diffractometer	2806 independent reflections
Radiation source: fine-focus sealed tube	2407 reflections with *I* > 2σ(*I*)
graphite	*R*_int_ = 0.051
Detector resolution: 8.33 pixels mm^-1^	θ_max_ = 67.4°, θ_min_ = 3.2°
phi and ω scans	*h* = −9→9
Absorption correction: multi-scan (*SADABS*; Bruker, 2008)	*k* = −7→9
*T*_min_ = 0.454, *T*_max_ = 0.914	*l* = −16→16
15401 measured reflections	

### Refinement

**Table:**

Refinement on *F*^2^	Primary atom site location: structure-invariant direct methods
Least-squares matrix: full	Secondary atom site location: difference Fourier map
*R*[*F*^2^ > 2σ(*F*^2^)] = 0.043	Hydrogen site location: inferred from neighbouring sites
*wR*(*F*^2^) = 0.118	H atoms treated by a mixture of independent and constrained refinement
*S* = 1.03	*w* = 1/[σ^2^(*F*_o_^2^) + (0.0805*P*)^2^ + 0.1676*P*] where *P* = (*F*_o_^2^ + 2*F*_c_^2^)/3
2806 reflections	(Δ/σ)_max_ < 0.001
242 parameters	Δρ_max_ = 0.40 e Å^−^^3^
0 restraints	Δρ_min_ = −0.21 e Å^−^^3^

### Special details

**Table:**

Geometry. All e.s.d.'s (except the e.s.d. in the dihedral angle between two l.s. planes) are estimated using the full covariance matrix. The cell e.s.d.'s are taken into account individually in the estimation of e.s.d.'s in distances, angles and torsion angles; correlations between e.s.d.'s in cell parameters are only used when they are defined by crystal symmetry. An approximate (isotropic) treatment of cell e.s.d.'s is used for estimating e.s.d.'s involving l.s. planes.
Refinement. Refinement of *F*^2^ against ALL reflections. The weighted *R*-factor *wR* and goodness of fit *S* are based on *F*^2^, conventional *R*-factors *R* are based on *F*, with *F* set to zero for negative *F*^2^. The threshold expression of *F*^2^ > σ(*F*^2^) is used only for calculating *R*-factors(gt) *etc*. and is not relevant to the choice of reflections for refinement. *R*-factors based on *F*^2^ are statistically about twice as large as those based on *F*, and *R*- factors based on ALL data will be even larger.

### Fractional atomic coordinates and isotropic or equivalent isotropic displacement parameters (Å^2^)

**Table:**

	*x*	*y*	*z*	*U*_iso_*/*U*_eq_	
Cl1	0.24720 (6)	0.24063 (6)	0.05019 (3)	0.02974 (18)	
O1	0.8362 (2)	1.58116 (19)	−0.72107 (11)	0.0356 (4)	
O2	0.0147 (2)	0.5892 (2)	0.10617 (11)	0.0319 (4)	
N1	0.3212 (2)	0.6808 (2)	−0.09907 (12)	0.0286 (4)	
N2	0.5247 (2)	0.3608 (2)	−0.12489 (13)	0.0278 (4)	
N3	0.6421 (2)	0.9679 (2)	−0.39335 (12)	0.0289 (4)	
N4	0.7403 (2)	0.9508 (2)	−0.47300 (12)	0.0288 (4)	
C1	0.2241 (3)	0.8387 (3)	−0.08742 (15)	0.0301 (5)	
H1	0.1353	0.8451	−0.0320	0.036*	
C2	0.2451 (3)	0.9963 (3)	−0.15216 (16)	0.0307 (5)	
H2	0.1716	1.1059	−0.1405	0.037*	
C3	0.3723 (3)	0.9915 (3)	−0.23242 (15)	0.0289 (5)	
H3	0.3878	1.0975	−0.2771	0.035*	
C4	0.4799 (3)	0.8273 (3)	−0.24789 (14)	0.0265 (4)	
C5	0.6165 (3)	0.8060 (3)	−0.33052 (14)	0.0272 (4)	
C6	0.7152 (3)	0.6433 (3)	−0.34203 (15)	0.0281 (4)	
H6	0.8043	0.6327	−0.3970	0.034*	
C7	0.6860 (3)	0.4882 (3)	−0.27224 (15)	0.0277 (4)	
C8	0.7851 (3)	0.3169 (3)	−0.27964 (15)	0.0302 (5)	
H8	0.8755	0.3004	−0.3335	0.036*	
C9	0.7517 (3)	0.1734 (3)	−0.20927 (16)	0.0314 (5)	
H9	0.8194	0.0580	−0.2139	0.038*	
C10	0.6177 (3)	0.1993 (3)	−0.13131 (15)	0.0298 (5)	
H10	0.5928	0.1010	−0.0826	0.036*	
C11	0.5531 (3)	0.5072 (3)	−0.19183 (14)	0.0263 (4)	
C12	0.4470 (3)	0.6768 (3)	−0.17871 (14)	0.0258 (4)	
C13	0.7674 (3)	1.1138 (3)	−0.53279 (15)	0.0273 (4)	
C14	0.6952 (3)	1.2806 (3)	−0.50623 (16)	0.0337 (5)	
H14	0.6267	1.2879	−0.4441	0.040*	
C15	0.7232 (3)	1.4333 (3)	−0.56975 (16)	0.0344 (5)	
H15	0.6758	1.5458	−0.5508	0.041*	
C16	0.8206 (3)	1.4250 (3)	−0.66197 (15)	0.0302 (5)	
C17	0.8979 (3)	1.2599 (3)	−0.68827 (15)	0.0283 (4)	
H17	0.9682	1.2527	−0.7499	0.034*	
C18	0.8705 (3)	1.1070 (3)	−0.62302 (15)	0.0284 (4)	
H18	0.9236	0.9943	−0.6405	0.034*	
H4	0.902 (4)	1.565 (4)	−0.783 (2)	0.052 (8)*	
H19	0.442 (4)	0.377 (3)	−0.076 (2)	0.045 (7)*	
H20	0.077 (4)	0.494 (4)	0.091 (2)	0.054 (8)*	
H21	−0.055 (4)	0.622 (4)	0.062 (2)	0.050 (8)*	

### Atomic displacement parameters (Å^2^)

**Table:**

	*U*^11^	*U*^22^	*U*^33^	*U*^12^	*U*^13^	*U*^23^
Cl1	0.0299 (3)	0.0235 (3)	0.0305 (3)	0.00016 (18)	−0.00177 (19)	0.00097 (18)
O1	0.0425 (9)	0.0243 (8)	0.0323 (8)	−0.0014 (6)	0.0041 (7)	0.0007 (6)
O2	0.0383 (9)	0.0256 (8)	0.0288 (8)	0.0005 (6)	−0.0038 (7)	−0.0041 (6)
N1	0.0299 (9)	0.0255 (9)	0.0280 (9)	−0.0050 (7)	−0.0016 (7)	−0.0011 (7)
N2	0.0282 (9)	0.0249 (9)	0.0272 (9)	−0.0021 (7)	−0.0023 (7)	−0.0013 (7)
N3	0.0283 (9)	0.0280 (9)	0.0276 (9)	−0.0033 (7)	−0.0025 (7)	−0.0010 (7)
N4	0.0277 (9)	0.0292 (10)	0.0262 (9)	−0.0026 (7)	−0.0035 (7)	0.0005 (7)
C1	0.0291 (11)	0.0284 (11)	0.0298 (11)	−0.0026 (8)	0.0014 (8)	−0.0049 (8)
C2	0.0345 (11)	0.0226 (11)	0.0330 (11)	−0.0016 (8)	−0.0031 (9)	−0.0048 (8)
C3	0.0324 (11)	0.0233 (11)	0.0298 (11)	−0.0056 (8)	−0.0043 (8)	−0.0011 (8)
C4	0.0256 (10)	0.0268 (11)	0.0268 (10)	−0.0040 (8)	−0.0065 (8)	−0.0017 (8)
C5	0.0279 (10)	0.0253 (11)	0.0267 (10)	−0.0035 (8)	−0.0067 (8)	0.0005 (8)
C6	0.0268 (10)	0.0307 (11)	0.0251 (10)	−0.0040 (8)	−0.0005 (8)	−0.0040 (8)
C7	0.0261 (10)	0.0264 (11)	0.0290 (11)	−0.0014 (8)	−0.0049 (8)	−0.0034 (8)
C8	0.0286 (11)	0.0291 (11)	0.0309 (11)	−0.0008 (8)	−0.0024 (8)	−0.0059 (8)
C9	0.0321 (11)	0.0235 (11)	0.0358 (11)	−0.0001 (8)	−0.0045 (9)	−0.0033 (8)
C10	0.0321 (11)	0.0215 (11)	0.0337 (11)	−0.0022 (8)	−0.0067 (9)	−0.0002 (8)
C11	0.0277 (10)	0.0236 (10)	0.0274 (10)	−0.0047 (8)	−0.0067 (8)	−0.0014 (8)
C12	0.0254 (10)	0.0246 (11)	0.0269 (10)	−0.0032 (8)	−0.0049 (8)	−0.0032 (8)
C13	0.0258 (10)	0.0268 (11)	0.0271 (10)	−0.0022 (8)	−0.0054 (8)	−0.0001 (8)
C14	0.0363 (12)	0.0315 (12)	0.0280 (11)	−0.0025 (9)	0.0030 (9)	−0.0026 (9)
C15	0.0403 (12)	0.0244 (11)	0.0334 (12)	−0.0009 (9)	0.0024 (9)	−0.0040 (8)
C16	0.0306 (11)	0.0268 (11)	0.0300 (11)	−0.0031 (8)	−0.0046 (8)	0.0013 (8)
C17	0.0274 (10)	0.0286 (11)	0.0267 (10)	−0.0024 (8)	−0.0023 (8)	−0.0029 (8)
C18	0.0281 (10)	0.0266 (11)	0.0276 (10)	0.0007 (8)	−0.0053 (8)	−0.0025 (8)

### Geometric parameters (Å, °)

**Table:**

O1—C16	1.347 (2)	C6—C7	1.430 (3)
O1—H4	0.95 (3)	C6—H6	0.9500
O2—H20	0.84 (3)	C7—C11	1.404 (3)
O2—H21	0.85 (3)	C7—C8	1.408 (3)
N1—C1	1.327 (3)	C8—C9	1.376 (3)
N1—C12	1.355 (3)	C8—H8	0.9500
N2—C10	1.326 (3)	C9—C10	1.391 (3)
N2—C11	1.358 (3)	C9—H9	0.9500
N2—H19	0.87 (3)	C10—H10	0.9500
N3—N4	1.259 (2)	C11—C12	1.432 (3)
N3—C5	1.419 (3)	C13—C18	1.388 (3)
N4—C13	1.411 (3)	C13—C14	1.402 (3)
C1—C2	1.398 (3)	C14—C15	1.372 (3)
C1—H1	0.9500	C14—H14	0.9500
C2—C3	1.368 (3)	C15—C16	1.396 (3)
C2—H2	0.9500	C15—H15	0.9500
C3—C4	1.409 (3)	C16—C17	1.395 (3)
C3—H3	0.9500	C17—C18	1.384 (3)
C4—C12	1.405 (3)	C17—H17	0.9500
C4—C5	1.444 (3)	C18—H18	0.9500
C5—C6	1.362 (3)		
			
C16—O1—H4	112.0 (17)	C7—C8—H8	119.8
H20—O2—H21	103 (3)	C8—C9—C10	119.17 (19)
C1—N1—C12	116.57 (17)	C8—C9—H9	120.4
C10—N2—C11	123.09 (19)	C10—C9—H9	120.4
C10—N2—H19	120.1 (18)	N2—C10—C9	120.14 (19)
C11—N2—H19	116.8 (18)	N2—C10—H10	119.9
N4—N3—C5	115.16 (16)	C9—C10—H10	119.9
N3—N4—C13	113.91 (16)	N2—C11—C7	119.06 (18)
N1—C1—C2	123.65 (19)	N2—C11—C12	119.30 (18)
N1—C1—H1	118.2	C7—C11—C12	121.64 (18)
C2—C1—H1	118.2	N1—C12—C4	124.37 (18)
C3—C2—C1	119.52 (18)	N1—C12—C11	117.06 (17)
C3—C2—H2	120.2	C4—C12—C11	118.57 (18)
C1—C2—H2	120.2	C18—C13—C14	118.72 (19)
C2—C3—C4	119.12 (18)	C18—C13—N4	117.59 (18)
C2—C3—H3	120.4	C14—C13—N4	123.69 (18)
C4—C3—H3	120.4	C15—C14—C13	120.14 (19)
C12—C4—C3	116.77 (18)	C15—C14—H14	119.9
C12—C4—C5	119.30 (17)	C13—C14—H14	119.9
C3—C4—C5	123.92 (18)	C14—C15—C16	120.64 (19)
C6—C5—N3	124.55 (18)	C14—C15—H15	119.7
C6—C5—C4	121.23 (18)	C16—C15—H15	119.7
N3—C5—C4	114.16 (17)	O1—C16—C17	123.39 (19)
C5—C6—C7	120.71 (19)	O1—C16—C15	116.79 (18)
C5—C6—H6	119.6	C17—C16—C15	119.82 (19)
C7—C6—H6	119.6	C18—C17—C16	118.89 (19)
C11—C7—C8	118.16 (19)	C18—C17—H17	120.6
C11—C7—C6	118.55 (18)	C16—C17—H17	120.6
C8—C7—C6	123.28 (19)	C17—C18—C13	121.70 (18)
C9—C8—C7	120.38 (19)	C17—C18—H18	119.2
C9—C8—H8	119.8	C13—C18—H18	119.2
			
C5—N3—N4—C13	−178.33 (16)	C8—C7—C11—C12	179.75 (18)
C12—N1—C1—C2	0.2 (3)	C6—C7—C11—C12	0.2 (3)
N1—C1—C2—C3	−0.2 (3)	C1—N1—C12—C4	0.2 (3)
C1—C2—C3—C4	−0.2 (3)	C1—N1—C12—C11	179.76 (18)
C2—C3—C4—C12	0.6 (3)	C3—C4—C12—N1	−0.6 (3)
C2—C3—C4—C5	179.22 (19)	C5—C4—C12—N1	−179.33 (18)
N4—N3—C5—C6	13.2 (3)	C3—C4—C12—C11	179.85 (18)
N4—N3—C5—C4	−169.79 (17)	C5—C4—C12—C11	1.1 (3)
C12—C4—C5—C6	−0.7 (3)	N2—C11—C12—N1	−0.5 (3)
C3—C4—C5—C6	−179.33 (19)	C7—C11—C12—N1	179.51 (18)
C12—C4—C5—N3	−177.80 (17)	N2—C11—C12—C4	179.09 (17)
C3—C4—C5—N3	3.6 (3)	C7—C11—C12—C4	−0.9 (3)
N3—C5—C6—C7	176.79 (18)	N3—N4—C13—C18	−178.43 (17)
C4—C5—C6—C7	0.0 (3)	N3—N4—C13—C14	1.1 (3)
C5—C6—C7—C11	0.2 (3)	C18—C13—C14—C15	1.5 (3)
C5—C6—C7—C8	−179.3 (2)	N4—C13—C14—C15	−178.00 (19)
C11—C7—C8—C9	−0.3 (3)	C13—C14—C15—C16	1.2 (3)
C6—C7—C8—C9	179.22 (19)	C14—C15—C16—O1	177.6 (2)
C7—C8—C9—C10	0.7 (3)	C14—C15—C16—C17	−3.1 (3)
C11—N2—C10—C9	0.0 (3)	O1—C16—C17—C18	−178.53 (18)
C8—C9—C10—N2	−0.5 (3)	C15—C16—C17—C18	2.3 (3)
C10—N2—C11—C7	0.4 (3)	C16—C17—C18—C13	0.5 (3)
C10—N2—C11—C12	−179.60 (18)	C14—C13—C18—C17	−2.4 (3)
C8—C7—C11—N2	−0.3 (3)	N4—C13—C18—C17	177.18 (17)
C6—C7—C11—N2	−179.78 (17)		

### Hydrogen-bond geometry (Å, °)

**Table:**

*D*—H···*A*	*D*—H	H···*A*	*D*···*A*	*D*—H···*A*
O1—H4···O2^i^	0.95 (3)	1.65 (3)	2.586 (2)	166 (3)
N2—H19···Cl1	0.87 (3)	2.35 (3)	3.1077 (19)	145 (2)
O2—H20···Cl1	0.84 (3)	2.25 (3)	3.0959 (16)	180 (3)
O2—H21···Cl1^ii^	0.85 (3)	2.30 (3)	3.1467 (17)	170 (2)

Symmetry codes: (i) *x*+1, *y*+1, *z*−1; (ii) −*x*, −*y*+1, −*z*.
